# Analysis of Two-Player Quantum Games in an EPR Setting Using Clifford's Geometric Algebra

**DOI:** 10.1371/journal.pone.0029015

**Published:** 2012-01-18

**Authors:** James M. Chappell, Azhar Iqbal, Derek Abbott

**Affiliations:** 1 School of Chemistry and Physics, University of Adelaide, Adelaide, South Australia, Australia; 2 School of Electrical and Electronic Engineering, University of Adelaide, Adelaide, South Australia, Australia; University of Nottingham, United Kingdom

## Abstract

The framework for playing quantum games in an Einstein-Podolsky-Rosen (EPR) type setting is investigated using the mathematical formalism of geometric algebra (GA). The main advantage of this framework is that the players' strategy sets remain identical to the ones in the classical mixed-strategy version of the game, and hence the quantum game becomes a proper extension of the classical game, avoiding a criticism of other quantum game frameworks. We produce a general solution for two-player games, and as examples, we analyze the games of Prisoners' Dilemma and Stag Hunt in the EPR setting. The use of GA allows a quantum-mechanical analysis without the use of complex numbers or the Dirac Bra-ket notation, and hence is more accessible to the non-physicist.

## Introduction

Although its origins can be traced to earlier works [1]–[4], the extension of game theory [5], [6] to the quantum regime [7] was proposed by Meyer [8] and Eisert et al [9] and has since been investigated by others [10]–[48]. Game theory is a vast subject but many interesting strategic interactions can still be found in simple-to-analyze two-player two-strategy non-cooperative games. The well known games of Prisoners' Dilemma (PD) and Stag Hunt [5], [6] are two such examples.

The general idea in the quantization scheme proposed by Eisert et al [9] for such games involves a referee who forwards a two-qubit entangled state to the two players. Players perform their strategic actions on the state that consist of local unitary transformations to their respective qubits. The qubits are subsequently returned to the referee for measurement from which the players' payoffs are determined. The setup ensures that players sharing a product initial state corresponds to the mixed-strategy version of the considered classical game. However, players sharing an entangled state can lead to new Nash equilibria (NE) [5], [6] consisting of pairs of unitary transformations [7], [9]. At these quantum NE the players can have higher payoffs relative to what they obtain at the NE in the mixed-strategy version of the classical game.

This approach to constructing quantum games was subsequently criticized [12] as follows. The players' strategic actions in the quantum game are extended operations relative to their actions in the original mixed-strategy version of the classical game, in which, each player can perform a strategic action consisting of a probabilistic combination of their two pure strategies. The mentioned criticism [12] argued that as the quantum players have expanded strategy sets and can do more than what the classical players can do, it is plausible to represent the quantum game as an extended classical game that also involves new pure strategies. The entries in the extended game matrix can then be suitably chosen so to be representative of the players' payoffs at the obtained quantum NE. This line of reasoning can be extended further in stating that quantum games are in fact ‘disguised’ classical games and to quantize a game is equivalent to replacing the original game by an extended classical game.

**Figure 1:**
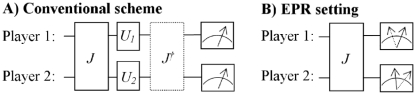
The EPR setting for playing quantum games compared with the conventional scheme. In the conventional scheme two qubits are entangled using an entangling operator 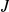, after which each player applies a unitary transformation 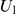,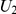 on their respective qubits. The supervisor then applies the inverse entangling operation (some researchers omit this operation) followed by measurement with Stern-Gerlach detectors. The EPR scheme, on the other hand, while it creates a general entangled state, each player is simply presented with a classical choice between two possible measurement directions for their Stern-Gerlach detector, as represented by the two arrows, so that the players strategy sets remain classical.

As a way to counter the criticism in [12], two-party Einstein-Podolsky-Rosen (EPR) type experiments [49]–[56] are recognized to have genuinely quantum features. One observes that the setting of such experiments can be fruitfully adapted [25], [28], [34], [42], [45] for playing a quantum version of a two-player two-strategy game, which allows us to avoid the criticism from another perspective. In particular, with the EPR type setting the players' strategies can be defined entirely classically–consisting of a probabilistic combination of a player's choice between two measurement directions. That is, with this setting, the players' strategy sets remain identical to ones they have in a standard arrangement for playing a mixed-strategy version of a classical two-player two-strategy game. As the players' strategy sets in the quantum game are not extended relative to the classical game, for this route to constructing quantum games, the mentioned criticism [12] does not apply. A diagram comparing quantum games in an EPR setting with a conventional quantum game setup is shown in Fig. 1.

The usefulness of applying the formalism of geometric algebra (GA) [57]–[63] in the investigation of quantum games has recently been shown [46] for the well known quantum penny flip game [8]. One may ask about the need of using the formalism of GA when, for instance, the GA based analysis of two-player quantum games developed in the following can also be reproduced with the standard analysis with Pauli matrices. We argue that the Pauli matrices are not always the preferred representation. Especially, as it is quite often overlooked that the algebra of Pauli matrices is the matrix representation for the Clifford's geometric algebra 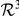, which is no more and no less than a system of directed numbers representing the geometrical properties of Euclidean 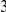-space. As a GA based analysis allows using operations in 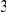-space with *real coordinates*, it thus permits a visualization that is simply not available in the standard approach using matrices over the field of complex numbers. Pauli matrices are isomorphic to the quaternions, and hence represent rotations of particle states. This fact paves the way to describe general unitary transformations on qubits, in a simplified algebraic form, as *rotors* that bring noticeable simplifications and geometrical clarifications. We apply constraints on the parameters of EPR type arrangements that ensure a faithful embedding of the mixed-strategy version of the original classical game within the corresponding quantum game. In particular, we show how using GA we can determine new NE in quantum games of Stag Hunt and Prisoners' Dilemma played in the EPR type setting.

### EPR setting for playing a quantum game

We have the following payoff matrices
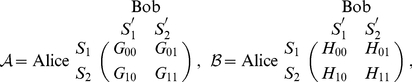
(1)giving Alice's and Bob's payoffs, respectively. Here Alice's pure strategies are 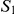 and 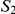 and Bob's pure strategies are 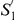 and 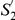. In a run, Alice chooses her strategy to be either 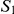 or 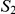 and likewise, in the same run, Bob chooses his strategy to be either 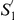 or 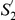. We consider games with symmetrical payoffs for which 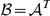, where 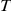 indicates transpose. This requires 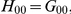 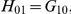 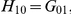 and 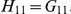


The EPR setting assumes that players Alice and Bob are spatially-separated participants, who are located at the two arms of the EPR system. In a run, each player receives one half of a two-particle system emitted by the same source. We associate Alice's strategies 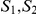 to the directions 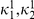 respectively and similarly, associate Bob's strategies 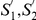 to the directions 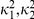, respectively. On receiving a pair of particles, players Alice and Bob together choose a pair of directions from the four possible cases 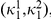 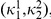 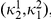 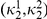 and a quantum measurement is performed along the chosen pair. The outcome of the measurement at either arm is 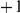 or 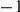. Over a large number of runs, a record is maintained of the players' choices of directions, representing their strategies, and one of the four possible outcomes 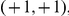 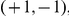 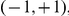 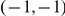 emerging out of the measurement. Within each of the brackets, the first entry is reserved for the outcome at Alice's side and the second entry for the outcome at Bob's side. Players' payoff relations are expressed in terms of the outcomes of measurements that are recorded for a large number of runs, as the players sequentially receive, two-particle systems emitted from the source. These payoffs depend on the strategic choices that each player adapts for his/her two directions over many runs, and on the dichotomic outcomes of the measurements performed along those directions. We specify that player payoffs are to be determined over a larger number of runs, because in this setup the directions of measurements are defined as players' strategies and for one set of directions (strategies) the measurement returns one of the four possible probabilistic outcomes 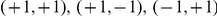, and 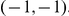 In classical game theory a given pair of players' strategies uniquely determines the payoff for each player but a single run in an EPR experiment cannot uniquely determine players' payoffs as for the same strategies (directions) their is still a probabilistic outcome arising from the nature of the measurement of quantum states.

### Geometric algebra

Geometric algebra (GA) [57]–[61] is an associative non-commutative algebra, that can provide an equivalent description to the conventional Dirac bra-ket and matrix formalisms of quantum mechanics, consisting of solely of algebraic elements over a strictly real field. Recently, Christian [64], [65] has used the formalism of GA in thought provoking investigations of some of the foundational questions in quantum mechanics. In the area of quantum games, GA has been used by Chappell et al [46] to determine all possible unitary transformations that implement a winning strategy in Meyer's PQ penny flip quantum game [8], and also in analyzing three-player quantum games [48].

Given a linear vector space 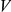 with elements 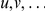 we may form [66] the tensor product 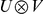 of vector spaces 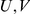, containing elements (bivectors) 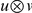 and hence construct the exterior or wedge product 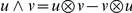. This may be extended to a vector space 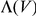 with elements consisting of multivectors that can be multiplied by means of the exterior product. The geometric product 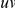 of two vectors 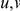 is defined by 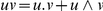, where 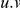 is the scalar inner product. The geometric product is in general not commutative though it is always associative, i.e. 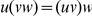.

We denote by 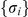 an orthonormal basis in 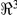, then 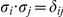. We also have 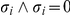 for each 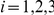 and so in terms of the geometric product we have 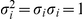, and 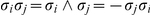 for each 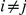. Hence the basis vectors anticommute with respect to the geometric product. If we denote by 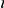 the trivector
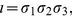
(2)then for distinct basis vectors we have
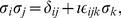
(3)where 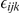 is the Levi-Civita symbol. We find that 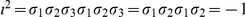 and commutes with all other elements and so has identical properties to the conventional complex number 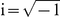. Thus we have an isomorphism between the basis vectors 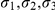 and the Pauli matrices through the use of the geometric product.

In order to express quantum states in GA we use the one-to-one mapping [59]–[61] defined as follows
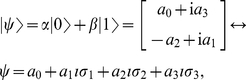
(4)where 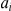 are real scalars.

It can then be shown using the Schmidt decomposition of a general two qubit state [61], that a general two-particle state can be represented in GA as
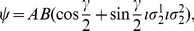
(5)where 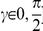 is a measure of the entanglement and where 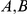 are single particle rotors applied to the first and second qubit, respectively. General unitary operations are called [59] rotors in GA, represented as
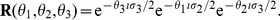
(6)This rotation, in Euler angle form, can completely explore the available space of a single qubit, and is equivalent to a general unitary transformation acting on a spinor. So, we have the rotors for each qubit defined as
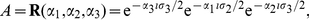
(7)

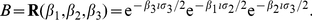
(8)For example, for 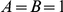 and 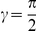, we find the Bell state, and 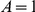 and 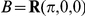 and 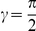 we recover the singlet state. This can be checked using Eq. (4), where we note that 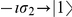.

To simulate the process of measurement in GA, we form a separable state 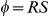, where 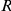 and 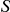 are single particle rotors, which allow general measurement directions to be specified, on the first and second qubit respectively. The state to be measured is now projected onto the separable state 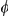. In the 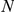-particle case, the probability that the quantum state 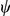 returns the separable state 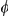 is given is Ref. [50] as
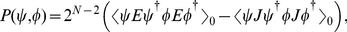
(9)where the angle brackets 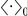 mean to retain only the scalar part of the expression. As noted by Doran, ‘Expressions such as this are unique to the geometric algebra approach’ [59]. We have the two observables 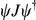 and 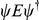, which in the two particle case involves [59]

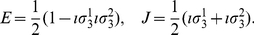
(10)The 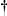 operator is analogous to complex conjugation, flipping the sign of 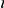 and inverting the order of terms. The measurement outcomes given by 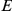 and 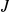 relate to standard quantum mechanics observables as follows:
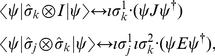
(11)where 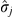 are the standard Pauli matrices [59].

## Results

Employing Eq. (9), we firstly calculate
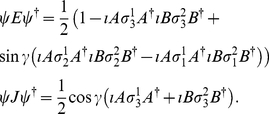
(12)To describe the players measurement directions, we have 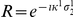 and 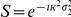. For the quantum game in the EPR setting, 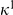 can be either of Alice's two directions i.e. 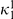 or 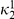. Similarly, in the expression for 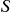 the 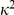 can be either of Bob's two directions i.e. 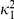 or 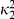. Hence we obtain
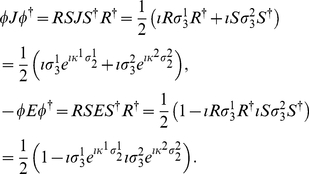
(13)Now from Eq. (9), we calculate
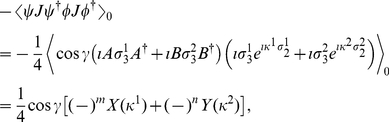
(14)where 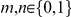 refers to measuring a 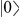 or a 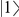 state, respectively, and using Eq. (49) we have
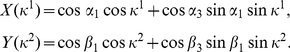
(15)Also, from Eq. (9) we obtain
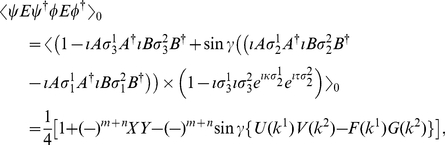
(16)where
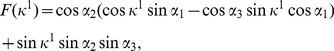
(17)

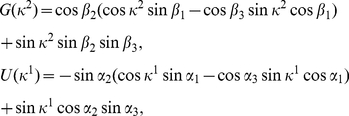
(18)

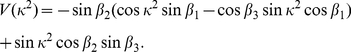
(19)Now combining Eq. (14) and Eq. (16), where we define 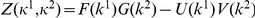, we have the probability to observe a particular state
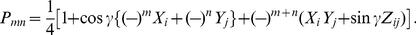
(20)To simplify notation we have written 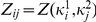 , 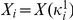 and 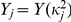, where 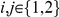 represent the two possible measurement directions available to each player. If we put 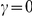, that is, for no entanglement, we have the probability
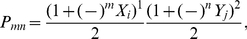
(21)which shows a product state incorporating general measurement directions for each qubit. This formula for 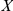 and 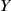 in Eq. 15 can be given a geometric interpretation as the projection of the polarization axis of a qubit, as envisaged on the Bloch sphere, onto the measurement plane 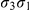 (based on the definition of the measurement rotor given earlier as 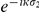). For example as a special case, with 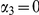, we have from Eq. (15) that 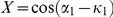, which is simply the difference in angle between the polarization axis and measurement axis. The case with two entangled qubits is more complex, as not just the initial polarization axis 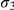, but also the axes 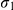 and 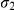 of each qubit effect the measurement outcome in a non-trivial manner. It has been shown that two qubits can described in a real 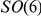 space using geometric algebra, and entangling operations involve rotating planes within this space [67].

### Finding the payoff relations

We allow each player the classical probabilistic choice between their two chosen measurement directions for their Stern-Gerlach detectors. The two players, Alice and Bob choose their first measurement direction with probability 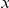 and 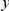 respectively, where 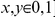. Now, we have the mathematical expectation of Alice's payoff, where she chooses the direction 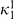 with probability 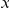 and the measurement direction 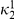 with probability 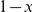, as
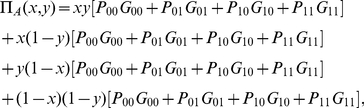
(22)where we have used the payoff matrix, defined for Alice, in Eq. (1) and the subscript 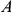 refers to Alice. We also define
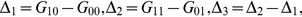
(23)so that by using Eqs. (20) the payoff for Alice (22) is expressed as
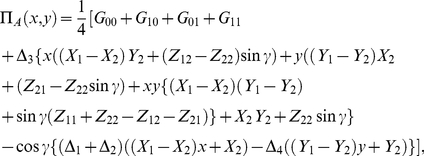
(24)where 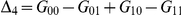. Bob's payoff, when Alice plays 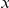 and Bob plays 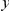 can now be obtained by interchanging 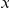 and 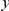 in the right hand side of Eq. (24).

### Solving the general two-player game

We now find the optimal solutions by calculating the Nash equilibrium (NE), that is, the expected response assuming rational self interest. To find the NE we simply require
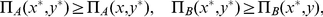
(25)which is stating that any unilateral movement of a player away from the NE of 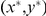, will result in a lower payoff for that player. We find
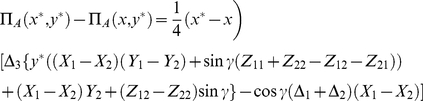
(26)and for the second player Bob we have similarly
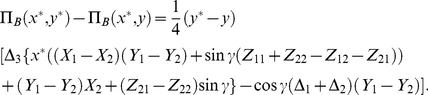
(27)


### Embedding the classical game

To embed the classical game, we require at zero entanglement, not only the same pair of strategies being a NE but also to have the bilinear structure of the classical payoff relations. At a NE of 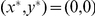, with zero entanglement, we find the payoff from Eq. (24) to be
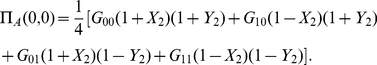
(28)This result illustrates how we could select any one of the payoff entries we desire with the appropriate selection of 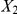 and 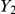, however in order to achieve the classical payoff of 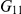 for this NE, we can see that we require 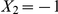 and 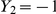. If we have a game which also has a classical NE of 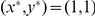 then from Eq. (24) at zero entanglement we find the payoff
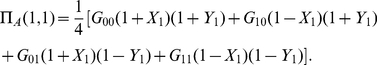
(29)So, we can see, that we can select the required classical payoff, of 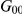, by the selection of 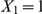 and 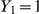.

Referring to Eq. (15), we then have the conditions
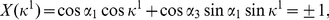
(30)

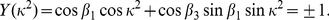
(31)Looking at the equation for Alice, we have two classes of solution: If 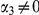, then for the equations satisfying 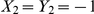, we have for Alice in the first equation 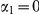, 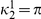 or 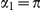, 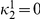 and for the equations satisfying 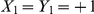, we have 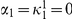 or 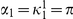, which can be combined to give either 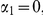 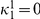 and 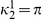 or 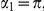 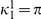 and 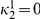. For the second class with 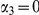, we have the solution 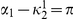 and for 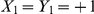 we have 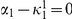.

So, in summary, for both cases we have that the two measurement directions are 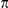 out of phase with each other, and for the first case (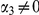) we can freely vary 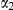 and 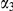, and for the second case (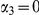), we can freely vary 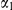 and 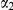 to change the initial quantum quantum state without affecting the game NE or the payoffs. The same arguments hold for the equations for 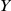. Combining these results and substituting into Eq. (19), we find that
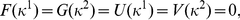
(32)and hence that
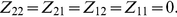
(33)


This then reduces the equation governing the NE in Eq. (26) to
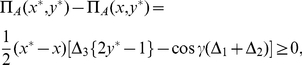
(34)which now has the new quantum behavior governed solely by the entanglement angle 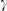. We have the associated payoffs
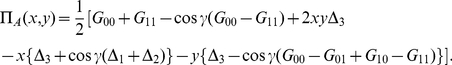
(35)Setting 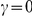 in Eq. (35) we find
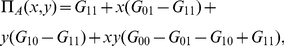
(36)which has the classical bilinear payoff structure in terms of 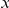 and 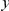. Hence we have faithfully embedded the classical game inside a quantum version of the game, when the entanglement goes to zero.

We also have the probabilities for each state 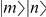, after measurement from Eq. (20), for this form of the quantum game as
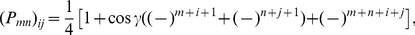
(37)for the two measurement directions 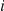 and 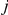.

### Examples

Here we explore the above results for the games of Prisoners' Dilemma and Stag Hunt. The quantum versions of these games are discussed in Refs. [9], [11], [19], [20], [24], [44].

#### Prisoners' Dilemma

The game of Prisoners' Dilemma (PD) [6] is widely known to economists, social and political scientists and is one of the earliest games to be investigated in the quantum regime [9]. Prisoner dilemma describes the following situation: two suspects are investigated for a crime that authorities believe they have committed together. Each suspect is placed in a separate cell and may choose between not confessing or confessing to have committed the crime. Referring to the matrices (1) we take 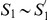 and 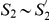 and identify 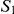 and 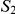 to represent the strategies of ‘not confessing’ and ‘confessing’, respectively. If neither suspect confesses, i.e. 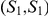, they go free, which is represented by 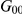 units of payoff for each suspect. The situation 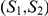 or 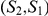 represents in which one prisoner confesses while the other does not. In this case, the prisoner who confesses gets 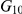 units of payoff, which represents freedom as well as financial reward as 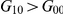, while the prisoner who did not confess gets 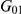, represented by his ending up in the prison. When both prisoners confess, i.e. 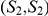, they both are given a reduced term represented by 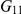 units of payoff, where 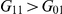, but it is not so good as going free i.e. 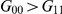.

With reference to Eq. (23), we thus have 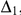 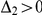. However, depending on the relative sizes of 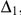 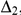 the quantity 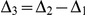 can be positive or negative. At maximum entanglement (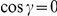), we note from Eq. (34), that there are two cases depending on 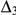. If 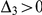, we notice that both the NE of 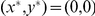 and 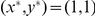 are present, and from Eq. (35) we have the payoff in both cases
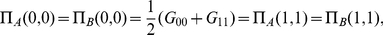
(38)which is a significant improvement over the classical payoff of 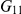. For 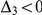, we have the two NE of 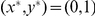 and 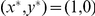, and from Eq. (35) we have the payoff
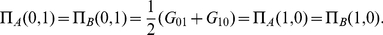
(39)If we reduce the entanglement of the qubits provided for the game, increasing 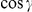 towards one, then from Eq. (34), we find a phase phase transition to the classical NE of 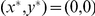, at 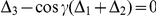 or
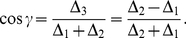
(40)Because we know that 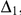 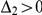, for the PD game, then a phase transition to the classical NE is guaranteed to occur, in the range 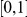.

**Figure 2:**
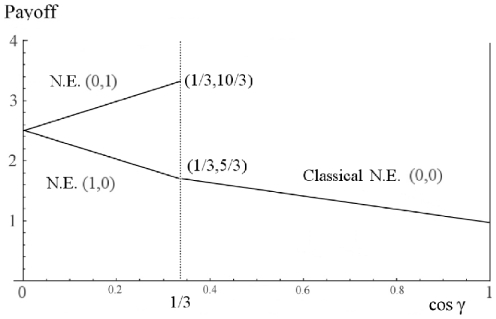
The PD game played in an EPR setting. We see that the classical equilibrium of 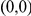 and the corresponding payoff of one unit is returned at zero entanglement (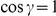). As the entanglement is increased, the payoff for each player increases until the entanglement reaches 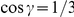 at which point there is a phase transition to new N.E of 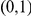 and 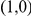. At maximum entanglement both players payoffs are equal at 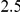 units, well above the classical payoff of one unit, and close to the Pareto optimal payoff of three units.

Consider a particular example of PD by taking 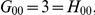 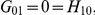 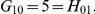 and 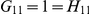 in matrices (1). From (23) we find 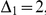 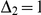 and 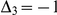 and we obtain 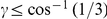 for a transition to the classical NE. Thus, for this PD game, to generate a non-classical NE the entanglement parameter 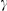 should be greater than 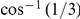. The new NE and payoffs can be calculated from Eq. (34) and Eq. (35) respectively, and refer to Fig. 2 for a diagram detailing these new NE and payoffs. For example the equation for the payoffs in the classical region (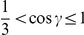) becomes 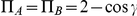.

#### Stag Hunt

The game of Stag Hunt (SH) [6] is encountered in the problems of social cooperation. For example, if two hunters are hunting for food, in a situation where they have two choices, either to hunt together and kill a stag, which provides a large meal, or become distracted and hunt rabbits separately instead, which while tasty, make a substantially smaller meal. Hunting a stag of course is quite challenging and the hunters need to cooperate with each other in order to be successful. The game of SH has three classical NE, two of which are pure and one is mixed. The two pure NE correspond to the situation where both hunters hunt the stag as a team or where each hunts rabbits by himself.

The SH game can be defined by the conditions 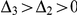 and 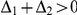 and 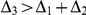. In the classical (mixed-strategy) version of this game three NE (two pure and one mixed) appear consisting of 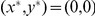, 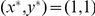 and 
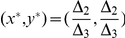
. From Eq. (34) and the defining conditions of SH game we notice that both the strategy pairs 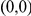 and 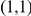 also remain NE in the quantum game for an arbitrary 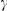. Eq. (35) give the players' payoffs at these NE as follows:
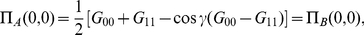
(41)

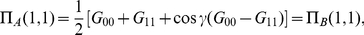
(42)which assume the values 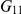 and 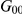 at 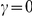, respectively. When 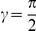 we have 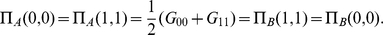 For the mixed NE for the quantum SH game we require from Eq. (34), 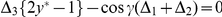 or
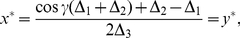
(43)which returns the classical mixed NE of 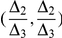 at zero entanglement. Depending on the amount of entanglement, the pair 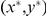, however, will shift themselves between 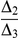 and 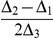. Players' payoffs at this shifted NE can be obtained from Eq. (35). Consider a particular example of SH by taking 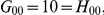 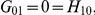 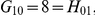 and 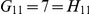 in matrices (1). From (23) we find 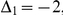 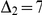 and 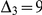. At 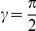 we have 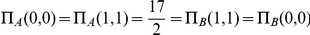. That is, the players' payoffs at the NE strategy pair 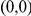 are increased from 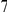 to 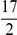 while at the NE strategy pair 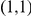 these are decreased from 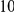 to 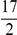. The mixed NE in the classical game is at 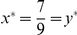 whereas it shifts to 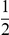 at 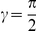.

## Discussion

The EPR type setting for playing a quantum version of a two-player two-strategy game is explored using the formalism of Clifford geometric algebra (GA), used for the representation of the quantum states, and the calculation of observables. We find that analyzing quantum games using GA comes with some clear benefits, for instance, improved perception of the quantum mechanical situation involved and particularly an improved geometrical visualization of quantum operations. To obtain equivalent results using the familiar algebra with Pauli matrices would be possible but obscures intuition. We also find that an improved geometrical visualization becomes helpful in significantly simplifying quantum calculations, for example unitary transformations on a single qubit become simply rotations of a vector as displayed on the Bloch sphere, and two qubits can be modeled in a real 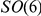 space [67] and we also find unique expressions in GA, such as Eq. (9) describing measurement outcomes for 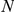 qubits.

We find that by using an EPR type setting we produce a faithful embedding of symmetric mixed-strategy versions of classical two-player two-strategy games into its quantum version, and that GA provides a simplified formalism over the field of reals for describing quantum states and measurements.

For a general two-player two-strategy game, we find the governing equation for a strategy pair forming a NE and the associated payoff relations. We find that at zero entanglement the quantum game returns the same pair(s) of NE as the classical mixed-strategy game, while the payoff relations in the quantum game reduce themselves to their bilinear form corresponding to a mixed-strategy classical game. We find that, within our GA based analysis, even though the requirement to properly embed a classical game puts constraints on the possible quantum states allowing this, we still have a degree of freedom, available with the entanglement angle 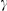, with which we can generate new NE. As a specific example the PD was found to have a NE of 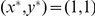 at high entanglement.

Analysis of quantum PD game in this paper can be compared with the results developed for this game in Ref. [34] also using an EPR type setting, directly from a set of non-factorizable joint probabilities. Although Ref. [34] and the present paper both use an EPR type setting, they use non-factorizability and entanglement for obtaining a quantum game, respectively. Our recent work [47] has observed that Ref. [34] does not take into consideration a symmetry constraint on joint probabilities that is relevant both when joint probabilities are factorizable or non-factorizable. When this symmetry constraint is taken into consideration, an analysis of quantum PD game played using an EPR setting does generate a non-classical NE in agreement with the results in this paper.

The EPR setting represents a simplified quantum game framework retaining classical strategies, but allowing quantum mechanical features such as entanglement to be employed in classical games. A more general scheme can be described allowing full use of unitary operations by each player, which is a useful framework when contact is not essential with a corresponding classical game. An even more general framework than quantum mechanics can be described, based on the properties of non-factorizable joint probabilities [47].

### Analysis

#### Calculating the observables

These three results are useful when calculating measurement outcomes in an EPR experiment, with a measurement direction 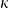, with a qubit defined by a rotor
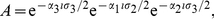
(44)and for measurement we use a rotor
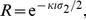
(45)defining rotations in the plane. We evaluate the quantities 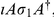 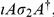 and 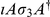 as follows.
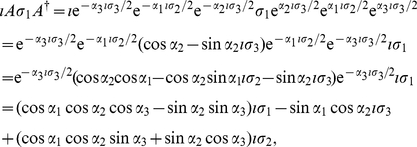
(46)

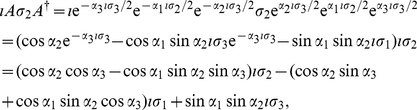
(47)

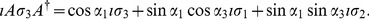
(48)We thus find for a general measurement direction 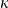, the following results
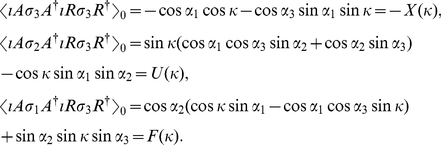
(49)


## References

[1] Blaquiere A (1980). Wave mechanics as a two-player game.. Dynamical Systems and Microphysics.

[2] Wiesner S (1983). Conjugate coding.. ACM Sigact News.

[3] Mermin N (1990). Quantum mysteries revisited.. American Journal of Physics.

[4] Mermin N (1990). Extreme quantum entanglement in a superposition of macroscopically distinct states.. Physical Review Letters.

[5] Binmore K (2007). Game theory: a very short introduction, volume 173..

[6] Rasmusen E (2007). Games and information: An introduction to game theory.

[7] Peres A (1993). Quantum theory: concepts and methods, volume 57.

[8] Meyer D (1999). Quantum strategies.. Physical Review Letters.

[9] Eisert J, Wilkens M, Lewenstein M (1999). Quantum games and quantum strategies.. Physical Review Letters.

[10] Vaidman L (1999). Time-symmetrized counterfactuals in quantum theory.. Foundations of physics.

[11] Benjamin S, Hayden P (2001). Multiplayer quantum games.. Physical Review A.

[12] van Enk SJ, Pike R (2002). Classical rules and quantum games.. Phys Rev A.

[13] Johnson N (2001). Playing a quantum game with a corrupted source.. Phys Rev A.

[14] Marinatto L, Weber T (2000). A quantum approach to games of static information.. Phys Lett A.

[15] Iqbal A, Toor A (2001). Evolutionarily stable strategies in quantum games.. Physics Letters A.

[16] Du J, Li H, Xu X, Zhou X, Han R (2002). Entanglement enhanced multiplayer quantum games.. Physics Letters A.

[17] Du J, Li H, Xu X, Shi M, Wu J (2002). Experimental realization of quantum games on a quantum computer.. Physical Review Letters.

[18] Piotrowski E, Sadkowski J (2002). Quantum market games.. Physica A: Statistical Mechanics and its Applications.

[19] Flitney A, Abbott D (2003). Advantage of a quantum player over a classical one in 2×2 quantum games.. Royal Society of London Proceedings Series A.

[20] Flitney AP, Abbott D (2002). An introduction to quantum game theory.. Fluctuation and Noise Letters.

[21] Iqbal A, Toor A (2002). Backwards-induction outcome in a quantum game.. Physical Review A.

[22] Piotrowski E, Sladkowski J (2003). An invitation to quantum game theory.. International Journal of Theoretical Physics.

[23] Shimamura J, Ozdemir S, Morikoshi F, Imoto N (2004). Entangled states that cannot reproduce original classical games in their quantum version.. Physics Letters A.

[24] Flitney A, Abbott D (2005). Quantum games with decoherence.. Journal of Physics A: Mathematical and General.

[25] Iqbal A, Weigert S (2004). Quantum correlation games.. Journal of Physics A: Mathematical and General.

[26] Mendes R (2005). The quantum ultimatum game.. Quantum Information Processing.

[27] Cheon T, Tsutsui I (2006). Classical and quantum contents of solvable game theory on Hilbert space.. Physics Letters A.

[28] Iqbal A (2005). Playing games with EPR-type experiments.. Journal of Physics A: Mathematical and General.

[29] Nawaz A, Toor A (2004). Generalized quantization scheme for two-person non-zero sum games.. Journal of Physics A: Mathematical and General.

[30] Cheon T (2006). Game theory formulated on hilbert space.. Quantum Computing: Back Action.

[31] Shimamura J, Özdemir S, Morikoshi F, Imoto N (2004). Quantum and classical correlations between players in game theory.. International Journal of Quantum Information.

[32] Ichikawa T, Tsutsui I (2007). Duality, phase structures, and dilemmas in symmetric quantum games.. Annals of Physics.

[33] Özdemir S, Shimamura J, Imoto N (2007). A necessary and suffIcient condition to play games in quantum mechanical settings.. New Journal of Physics.

[34] Iqbal A, Cheon T (2007). Constructing quantum games from nonfactorizable joint probabilities.. Physical Review E.

[35] Ramzan M, Nawaz A, Toor A, Khan M (2008). The effect of quantum memory on quantum games.. Journal of Physics A: Mathematical and Theoretical.

[36] Flitney A, Hollenberg L (2007). Nash equilibria in quantum games with generalized two-parameter strategies.. Physics Letters A.

[37] Aharon N, Vaidman L (2008). Quantum advantages in classically defined tasks.. Physical Review A.

[38] Bleiler S (2008). A formalism for quantum games and an application..

[39] Ahmed A, Bleiler S, Khan F (2008). Three player, two strategy, maximally entangled quantum games..

[40] Guo H, Zhang J, Koehler G (2008). A survey of quantum games.. Decision Support Systems.

[41] Ichikawa T, Tsutsui I, Cheon T (2008). Quantum game theory based on the Schmidt decomposition.. Journal of Physics A: Mathematical and Theoretical.

[42] Iqbal A, Cheon T, Abbott D (2008). Probabilistic analysis of three-player symmetric quantum games played using the Einstein-Podolsky-Rosen-Bohm setting.. Physics Letters A.

[43] Li Q, He Y, Jiang J (2009). A novel clustering algorithm based on quantum games.. Journal of Physics A: Mathematical and Theoretical.

[44] Iqbal A, Abbott D (2009). Non-factorizable joint probabilities and evolutionarily stable strategies in the quantum prisoner's dilemma game.. Physics Letters A.

[45] Iqbal A, Abbott D (2009). Quantum matching pennies game.. Journal of the Physical Society of Japan.

[46] Chappell J, Iqbal A, Lohe M, Von Smekal L (2009). An analysis of the quantum penny ip game using geometric algebra.. Journal of the Physical Society of Japan.

[47] Chappell J, Iqbal A, Abbott D (2010). Constructing quantum games from symmetric nonfactorizable joint probabilities.. Physics Letters A.

[48] J M Chappell AI, Lohe MA (2011). Analyzing three-player quantum games in an EPR type setup.. PLoS ONE.

[49] Einstein A, Podolsky B, Rosen N (1935). Can quantum-mechanical description of physical reality be considered complete?. Physical review.

[50] Bohm D (1951). Quantum theory.

[51] Bell J (1964). On the Einstein-Podolsky-Rosen paradox.. Physics.

[52] Bell J (1987). Speakable and Unspeakable in Quantum Mechanics.

[53] Bell J (1966). On the problem of hidden variables in quantum mechanics.. Reviews of Modern Physics.

[54] Aspect A, Dalibard J, Roger G (1982). Experimental test of Bell's inequalities using time-varying analyzers.. Physical Review Letters.

[55] Clauser J, Shimony A (1978). Bell's theorem. experimental tests and implications.. Reports on Progress in Physics.

[56] Cereceda J (2000). Quantum mechanical probabilities and general probabilistic constraints for Einstein- Podolsky- Rosen- Bohm experiments.. Foundations of Physics Letters.

[57] Hestenes D (1999). New foundations for classical mechanics: Fundamental Theories of Physics.

[58] Hestenes D, Sobczyk G (1984). Clifford Algebra to Geometric Calculus: A unified language for mathematics and physics, volume 5.

[59] Doran C, Lasenby A (2003). Geometric Algebra for Physicists.

[60] De Sabbata V, Datta B (2007). Geometric Algebra and Applications to Physics.

[61] Parker R, Doran C (2001). Analysis of 1 and 2 particle quantum systems using geometric algebra..

[62] Chappell J, Iqbal A, Abbott D (2011). Geometric algebra: A natural representation of three-space..

[63] Chappell J, Iannella N, Iqbal A, Abbott D (2011). Revisiting special relativity: A natural algebraic alternative to Minkowski spacetime..

[64] Christian J (2007). Disproof of Bell's theorem by Clifford algebra valued local variables..

[65] Christian J (2011). Restoring local causality and objective reality to the entangled photons..

[66] Szekeres P (2004). A course in modern mathematical physics: groups, Hilbert space, and differential geometry.

[67] Havel T, Doran C (2004). A bloch-sphere-type model for two qubits in the geometric algebra of a 6-D Euclidean vector space..

